# Functionalized magnetic nanowires for chemical and magneto-mechanical induction of cancer cell death

**DOI:** 10.1038/srep35786

**Published:** 2016-10-24

**Authors:** Aldo Isaac Martínez-Banderas, Antonio Aires, Francisco J. Teran, Jose Efrain Perez, Jael F. Cadenas, Nouf Alsharif, Timothy Ravasi, Aitziber L. Cortajarena, Jürgen Kosel

**Affiliations:** 1 Division of Biological and Environmental Sciences and Engineering, King Abdullah University of Science and Technology, Thuwal Jeddah, 23955-6900, Saudi Arabia; 2 IMDEA Nanociencia and Nanobiotechnology Unit associated to Centro Nacional de Biotecnología (CNB-CSIC), Campus Universitario de Cantoblanco, Madrid, 28049, Spain; 3 CIC BiomaGUNE, Parque Tecnológico de San Sebastián, Paseo Miramón 182, Donostia-San Sebastián 20009, Spain; 4 Ikerbasque, Basque Foundation for Science, Mª Díaz de Haro 3, E-48013 Bilbao, Spain; 5 Division of Computer, Electrical and Mathematical Sciences and Engineering, King Abdullah University of Science and Technology, Thuwal Jeddah, 23955-6900, Saudi Arabia

## Abstract

Exploiting and combining different properties of nanomaterials is considered a potential route for next generation cancer therapies. Magnetic nanowires (NWs) have shown good biocompatibility and a high level of cellular internalization. We induced cancer cell death by combining the chemotherapeutic effect of doxorubicin (DOX)-functionalized iron NWs with the mechanical disturbance under a low frequency alternating magnetic field. (3-aminopropyl)triethoxysilane (APTES) and bovine serum albumin (BSA) were separately used for coating NWs allowing further functionalization with DOX. Internalization was assessed for both formulations by confocal reflection microscopy and inductively coupled plasma-mass spectrometry. From confocal analysis, BSA formulations demonstrated higher internalization and less agglomeration. The functionalized NWs generated a comparable cytotoxic effect in breast cancer cells in a DOX concentration-dependent manner, (~60% at the highest concentration tested) that was significantly different from the effect produced by free DOX and non-functionalized NWs formulations. A synergistic cytotoxic effect is obtained when a magnetic field (1 mT, 10 Hz) is applied to cells treated with DOX-functionalized BSA or APTES-coated NWs, (~70% at the highest concentration). In summary, a bimodal method for cancer cell destruction was developed by the conjugation of the magneto-mechanical properties of iron NWs with the effect of DOX producing better results than the individual effects.

Regardless of the continuous development and innovation in cancer therapy, cancer prevails at the top of death causes for adults as one of the most devastating diseases^1^,^2^. Current cancer treatment approaches such as surgery, radiation and chemotherapy are highly aggressive to the organism by their invasiveness and side effects. Additionally, the efficient use of chemical agents is affected by the development of the multidrug resistance phenotype in cancer cells^3^. Most pharmaceutical preparations have their primary targets within the cell; therefore selective subcellular delivery may increase the therapeutic efficiency and simultaneously overcome secondary effects. In this regard, nanotechnology may be used to achieve therapeutic dosing, establish sustained-release drug profiles^4^,^5^, and increase the half-life of drugs avoiding efflux or degradation^6^.

Nanomaterials possess novel structural, optical and electromagnetic properties and their pharmacokinetic parameters may be altered according to size, shape, and surface functionalization^4^. Their vast surface area provides them with the possibility of surface modifications for further conjugation of large amounts of therapeutic molecules such as targeting agents and anticancer drugs^3^,^7^,^8^. In addition, the potential of electromagnetic nanomaterials as a therapeutic agent arises from the intrinsic properties of the core combined with the biomedical properties generated by different surface coatings^9^. These surface modifications alter the pharmacokinetics and cytotoxicity in addition to enabling biomolecules attachment through covalent linkages^10^. The intrinsic properties of the core allow remote manipulation through the application of an electric or magnetic field. It has been observed that in the presence of an electric field, non-magnetic nanomaterials can be transported to specific locations^11^ while magnetic nanomaterials can be trapped, concentrated^12^,^13^,^14^,^15^, or used in cell separation^16^,^17^,^18^,^19^ by applying a magnetic field. Moreover, the influence of an alternating magnetic field (AMF) can induce heat^20^ or rotate the nanostructures^21^,^22^.

Most of the research done lately focused on magnetic nanoparticles (MNPs) and their development as a therapeutic option for cancer. It has been reported that magnetic nanowires (NWs) offer potential advantages over MNPs because of their larger surface area to volume ratio and higher magnetic moments originated from their strong shape anisotropy^23^,^24^. In the presence of an AMF, NWs can generate large forces and torques when trying to align to the magnetic moment with the applied AMF^24^,^25^. At low frequencies, a magneto-mechanical effect is produced by the oscillation of the NWs^25^ while a hyperthermia effect can be generated at high frequencies (~100 kHz)^26^,^27^. The large aspect ratio provides ferromagnetic NWs with large remanent magnetizations^28^, and hence they can be used in low-field environments, where MNPs do not perform at all^29^.

Several studies support the efficacy and utility of nickel (Ni) NWs in diverse applications such as cell separation, manipulation, and purification^17^,^18^,^23^,^24^,^30^,^31^,^32^,^33^, as well as in the delivery of cargos including biological entities^34^. Furthermore, they have been utilized as therapeutic agents for hyperthermia^35^ and induction of cell inflammation^36^ in cultures of human embryonic cells. Although a large amount of evidence place Ni as a good candidate material, important genotoxicity and cytotoxicity effects have also been reported for Ni-containing dust particles^37^. On the other hand, Fe NWs have shown a good biocompatibility even at high concentrations with long incubation periods^38^. A cross-comparison among studies^39^,^40^ implies that Fe NWs have a lower impact on cell viability than Ni NWs at a given concentration.

Internalization of NWs by cells has been documented over the past years for both Ni and Fe^41^. Regarding Ni NWs, it has been reported that the internalization takes place through the activation of the integrin-mediated phagocytosis pathway^24^,^42^, and finally degradated^43^. For Fe NWs two main uptake mechanisms were proposed, including non-specific pinocytosis for short NWs and perforation of the outer plasma membrane for longer NWs^39^. Likewise, after 24 hours of incubation with polymeric NWs made of iron oxide particles, it was observed that most of the NWs were located in the cytosol, with a small fraction located in late endosomal/lysosomal compartments, where they are probably broken in smaller pieces for further degradation due to the low pH^30^.

Studies of the alterations in cellular features due to magneto-mechanical effects from AMFs have been previously performed. A remarkable decrease of viability of dendritic cells loaded with MNPs was observed after 30 min of exposure to a hyperthermia-like AMF (16 mT, 260 kHz), without raising the temperature of the cell culture. Clear morphological changes including cell membrane leakage and cell shrinkage after magnetic field application were observed and adjudicated as the main reason for the cell death^44^. Recently, colon cancer cells were incubated with Ni NWs and exposed to a low frequency AMF (0.5 mT and 1 Hz) for 10 minutes, which exerted a force on the NWs, triggering a mechanical disturbance to the cells and therefore inducing cell death in a non-chemotoxic way. The temperature was monitored during the experiment and no differences were observed in comparison with the control group disregarding an increase in temperature as a contribution to reducing the viability of the cells^25^. Furthermore, Kossatz *et al*.^45^ have introduced a bimodal cancer therapy using MNPs by exploiting magnetic hyperthermia and chemotherapy. As a result, a synergic effect was produced, which translated in a strong enhancement of the cytotoxicity of the functionalized MNPs *in vitro* and *in vivo*^45^.

In this study, we introduce a new bimodal strategy for cancer cell death induction by combining the chemotherapeutic effect of doxorubicin (DOX)-functionalized Fe NWs with the mechanical disturbance exerted by them when a low frequency AMF is applied. The effectiveness of this approach was evaluated through the decrease in the viability of breast cancer cells.

## Results and Discussion

### Iron nanowires characterization and functionalization

**Figure 1:**
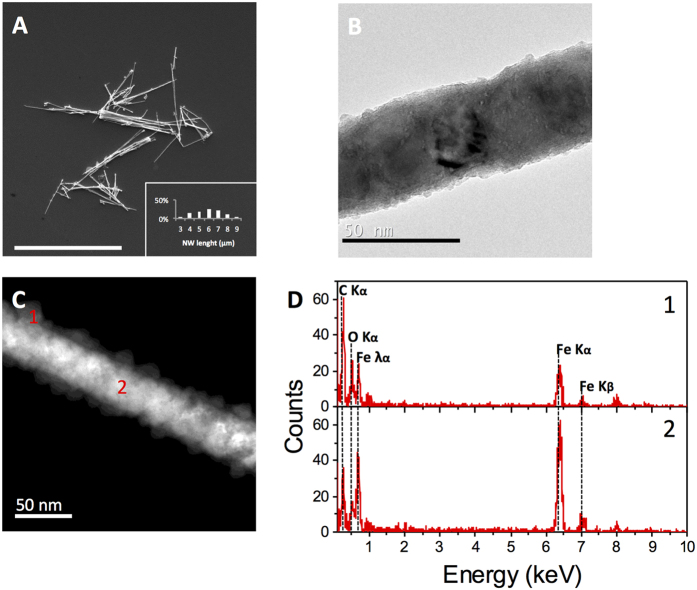
Characterization of Fe NWs. (**A**) SEM image of Fe NWs on top of a silicon wafer substrate, the inset corresponds to the NWs length distribution. Scale bar = 10 μm. (**B**) TEM image of a single Fe NW. Scale bar = 50 nm. (**C**) Scanning TEM image of a single Fe NW indicating its Fe_2_O_3_ surrounding layer (1) and core (2). Scale bar = 50 nm. D. Point EDX spectra showing the composition analysis of fabricated Fe NWs at its surrounding layer (1) and core (2).

Fe NWs were fabricated by electrodeposition onto alumina membranes produced by a two-step anodization process as explained in the Methods section. NWs were released from the alumina and their morphology (Fig. 1A,B) and chemical composition (Fig. 1C,D) were analyzed showing a core of solid Fe surrounded by an iron oxide (Fe_2_O_3_) interphase. The Fe NWs presented an average length of 6.4+/−1.3 μm and a diameter of 30 to 40 nm (Fig. 1A,B). Regardless of the synthesis method or morphology, metal particles will oxidize when exposed to air, oxygen, etc. Moreover, the use of NaOH during the release of NWs and ethanol during cleaning steps contributes to the NW oxidation. The Fe-oxide interphase has been previously characterized and was shown to be a thin layer of 4–10 nm on the surface of the NW^25^,^46^. Whereas polycrystalline Fe NWs completely oxidize over time, the thickness of the oxide shell of single-crystal Fe NWs does not increase beyond 10 nm, and the Fe core is maintained even at strong oxidizing conditions^46^. The Fe-oxide interphase has an important contribution to the biocompatibility^46^, functionalization^29^,^39^ and magnetic properties of the NWs. For the latter, magnetization measurements made recently revealed that the saturation and remanent magnetizations depend on the oxide interphase thickness^46^. In this regard, a reduction of 15% in the magnetization was observed for Fe NWs after 10 days in direct contact with air^47^.

**Figure 2:**
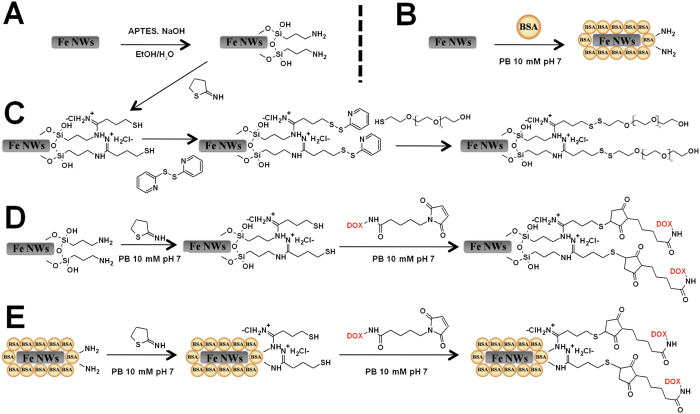
General scheme of Fe NWs coating and functionalization. (**A**) Coating of Fe NWs with APTES (APTES-NWs). (**B**) Coating of Fe NWs with BSA (BSA-NWs). (**C**) Coating of Fe NWs with APTES-PEG (APTES-PEG-NWs). (**D**) Functionalization of APTES-NWs with DOX (APTES-NWs-DOX). (**E**) Functionalization of BSA-NWs with DOX (BSA-NWs-DOX).

**Figure 3:**
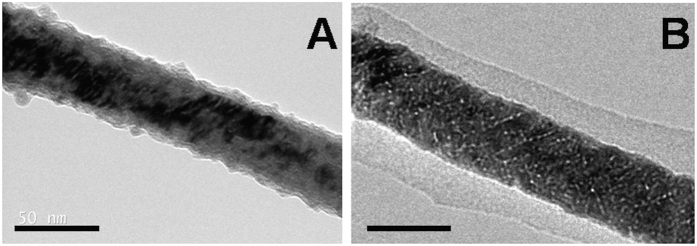
TEM micrographs of APTES-NWs (**A**) and BSA-NWs (**B**). The scale bars correspond to 50 nm.

As stated above, the Fe_2_O_3_ interphase allows the attachment of coating agents to the NW’s surface and thus, the Fe NWs were functionalized with three different biocompatible coatings, (3-aminopropyl)triethoxysilane (APTES), bovine serum albumin (BSA) and APTES-polyethylene glycol (PEG), to improve their stability under physiological conditions (Fig. 2). APTES and BSA were covalently attached to the NWs (APTES-NWs and BSA-NWs) by the reaction with the Fe_2_O_3_ interphase (Figs 2A,B and 3). A Bradford assay of the supernatant after the BSA coating process indicated an immobilization of 410 μg BSA/mg Fe. The presence of the BSA coating on the NWs’ surface was also verified by TEM imaging that showed a homogenous layer of protein around the NW (Fig. 3B).

The functionalization of APTES-NWs with PEG (APTES-PEG-NWs) was achieved by the formation of disulfide bonds between the reactive thiol of the thiol-PEG and the activated sulfhydryl groups of the modified APTES-NWs (Fig. 2C). The introduction of activated sulfhydryl groups onto the APTES-NWs was performed in two steps. First, free thiol groups were generated by the reaction between 2-iminothiolane (2-IT) and the amine groups of the APTES followed by the activation of the free thiol groups with aldrithiol. The process led to NWs bearing approximately 50 μmol of PEG/g Fe.

**Figure 4:**
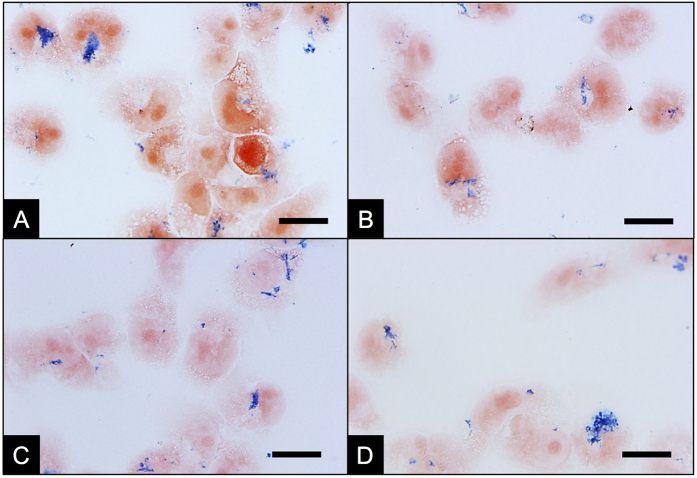
Interaction of Fe NWs coated with different agents and MDA-MB-231 cells 24 h post incubation using Prussian blue staining for iron oxide detection. (**A**) Fe NWs without coating. (**B**) APTES coated Fe NWs (APTES-NWs). (**C**) BSA coated Fe NWs (BSA-NWs). (**D**) APTES-PEG coated Fe NWs (APTES-PEG-NWs). Scale bar = 10 μm.

In order to qualitatively compare how the coating agents affect the interaction (internalization and/or attachment to the cellular surface) of the NWs and MDA-MB-231 breast cancer cells as well as their colloidal stability, a Prussian blue staining assay for Fe oxide detection was performed (Fig. 4). To this end, MDA-MB-231 breast cancer cells were incubated with the coated Fe NWs at 0.05 mg Fe/mL for 24 h, followed by several washes and the addition of a cyanide Fe salt. Finally, slides where prepared for bright field imaging (Supplementary Info). From the images in Fig. 4, a difference in the morphology, size and distribution of NW agglomerates can be observed between the different coatings, when compared to the non-coated NWs (Fig. 4A). Thereby, the three coating agents reduced the size of the agglomerates and increased the homogeneous distribution of the NWs across the sample. The effect is more pronounced for APTES-NWs and BSA-NWs (Fig. 4B,C) than for APTES-PEG-NWs (Fig. 4D). Furthermore, the oxidation state of Fe contained in the oxide interphase was corroborated by this assay as being Fe^3+^, as only Fe^3+^ would be able to react with the cyanide Fe salt Fe(CN)_6_^4−^, generating the blue coloration of the NWs observed in Fig. 4.

Based on these results, BSA-NWs and APTES-NWs were selected for further functionalization with DOX to evaluate their potential on cancer cell death induction by combining the chemotherapeutic effect with the magneto-mechanical one. The coated Fe NWs were functionalized with DOX through a pH-sensitive covalent bond to permit the selective drug release after internalization of the NWs. It has been assessed that covalent bonds sensible to acid hydrolysis are a suitable and efficient option for attaching drugs to magnetic nanoparticles due to the pH difference existing between the extra (pH 7.4) and intracellular compartments (pH 5.5–6.4 in endosomes)^3^,^48^,^49^,^50^. The functionalization was achieved by first introducing free thiol groups on the coated NWs by the reaction between 2-IT and the amine groups of the coated NWs (Fig. 2D,E). This process led to NWs bearing approximately 65 and 32 μmol of thiols/g Fe, for APTES- and BSA-NWs, respectively. Then, DOX was immobilized on the activated NWs by the reaction of the free thiols and the maleimide group of the DOX derivative (Fig. 2D,E). The final formulations contained 50 μmol DOX/g Fe in the case of APTES-NWs-DOX and 25 μmol DOXO/g Fe in the case of BSA-NWs-DOX.

Before performing *in vitro* cytotoxicity experiments, we studied the release of DOX from the functionalized NWs through the pH-sensitive covalent bond that allows the selective release of the unmodified drug in intracellular conditions. DOX was released from the DOX functionalized NWs after transferring them from PBS with pH 7.4 to acetate buffer with pH 5.0 (Supplementary Figure S1) in a time dependent manner. Fluorescence spectrophotometric measurements showed that 80% of DOX was released from APTES-NWs-DOX and BSA-NWs-DOX after 4 h, and reached a total release after 10 hours as shown in the release kinetics. In contrast, no appreciable release was observed in the control experiments where the functionalized NWs remained in PBS solution, which confirms the stability of the nanoformulations.

### Functionalized iron nanowires internalization and drug release monitoring

In order to assess the NWs internalization, ICP-MS measurements were carried out on cells incubated with NWs for 24 h, washed several times and lysed. The results showed no significant difference in the total amount of Fe from the cells incubated with APTES-NWs (31 ± 5 pg Fe/cell) and BSA-NWs (26 ± 3 pg Fe/cell). These results represent a total internalization of 19% and 15% for APTES-NWs and BSA-NWs, respectively. It is worth to mention that these values refer not only to internalized NWs, but also to NWs embedded or stuck in the extracellular structures surrounding the plasmatic membrane.

**Figure 5:**
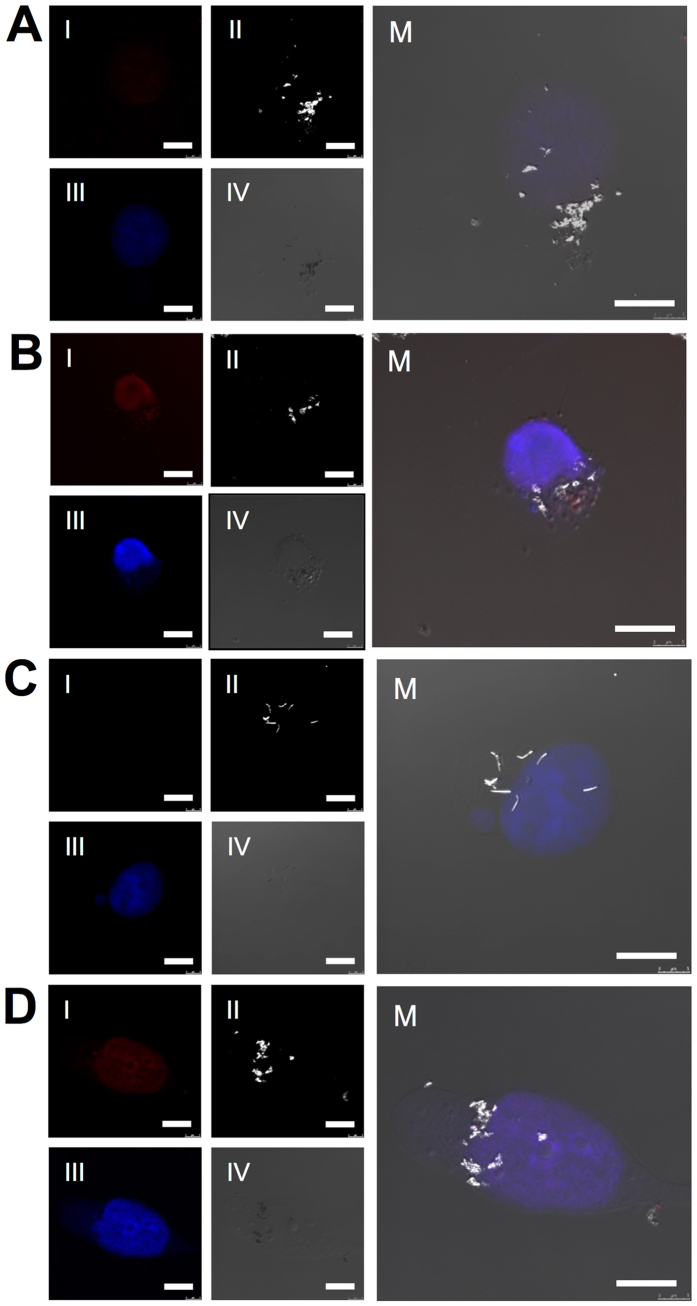
Confocal microscopy images from MDA-MB-231 cells incubated with APTES-NWs-DOX after 24 h (**A**) and after 72 h (**B**) and incubated with BSA-NWs-DOX after 24 h (**C**) and after 72 h (**D**). Four different channels: I. Red fluorescence of DOX, II. Light reflected from the Fe NWs, III. DAPI nuclear staining and IV. DIC. M. shows the merged image. Scale bars = 10 μm.

The NWs internalization and the selective intracellular drug release were evaluated through confocal microscopy studies. Images of intracellular focal planes were acquired after 24 and 72 h of incubation with APTES-NWs-DOX (Fig. 5A,B) and BSA-NWs-DOX (Fig. 5C,D). The red fluorescence of DOX permits its intracellular imaging. From Fig. 5 (panels I) the emergence of red fluorescence derived from the presence of DOX after 72 h (Fig. 5B,D) is evident when compared with 24 h (Fig. 5A,C). At 72 h the DOX fluorescence signal overlaps with the nucleus stained with DAPI (panels III), as shown in Fig. 5B,D. These results indicate the efficient release and nuclear localization of the chemotherapeutic agent. In all the conditions, functionalized NWs were observed as white structures, due to their capability of reflecting light in the reflection mode used to acquire the images (Fig. 5, panels II).

**Figure 6:**
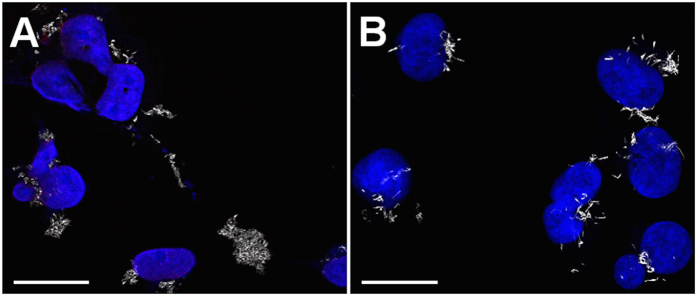
Morphology and distribution of functionalized NWs in MDA-MB-231 cells. (**A**) Cells treated with APTES-NWs-DOX after 24 h of incubation. (**B**) Cells treated with BSA-NWs-DOX after 24 h of incubation. Scale bars are 25 μm.

Furthermore, confocal intracellular Z-stacks from cells incubated 24 h with both formulations were generated to substantiate and compare the internalization of both NW formulations and are presented as separated images (Supplementary Figure S2) and stack-projections (Supplementary Figures S3 and S4). In all the conditions NWs were observed at the same focal plane as the nuclear staining, confirming their intracellular localization. A different distribution of the NWs is observed when comparing both NW formulations. This observation is perceived in greater detail in Fig. 6, which shows larger field images of an intracellular focal plane denoted by the presence of the nucleus. APTES-NWs-DOX (Fig. 6A) were less homogeneously distributed within the cell, being mainly located around the nucleus when compared to BSA-NWs-DOX (Fig. 6B), which distribute across the cell and appear in different focal planes of the Z-stack (Supplementary Figures S2–S4).

Morphological and quantitative differences were also perceived for both formulations. BSA-NWs-DOX agglomerated in less compact clusters and presented a needle-like shape while more compact clusters with a round shape were produced by APTES-NWs-DOX. Additionally, a larger intracellular amount of BSA-NWs-DOX was observed in Fig. 6, indicating a higher degree of internalization when compared to APTES-NWs-DOX.

### Bimodal strategy for cancer cell death induction with functionalized iron nanowires

**Figure 7:**
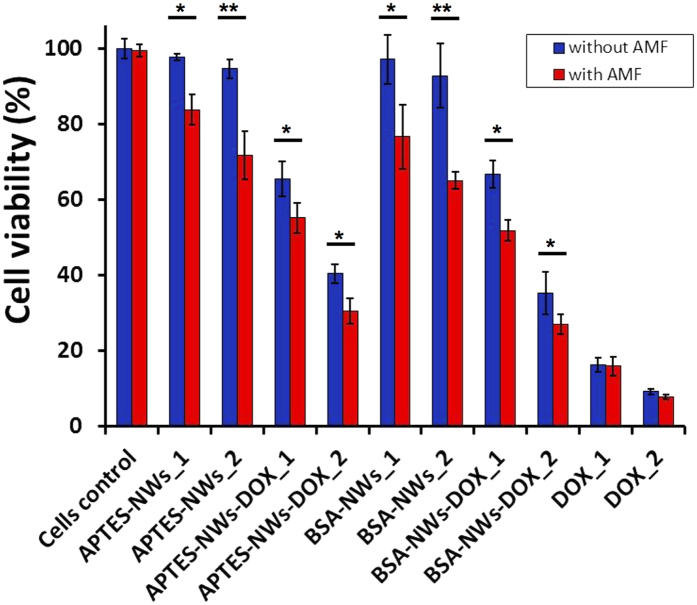
Viability of MDA-MB-231 cells incubated with different formulations and with or without application of a low frequency alternating magnetic field (AMF). APTES-NWs: 8.7 μg Fe/mL (APTES-NWs_1), 26 μg Fe/mL (APTES-NWs_2). APTES-NWs-DOX: 8.7 μg Fe/mL and 0.44 μM of DOX (APTES-NWs-DOX_1), 26 μg Fe/mL and 1.3 μM of DOX (APTES-NWs-DOX_2). BSA-NWs: 9.3 μg Fe/mL (BSA-NWs_1), 28 μg Fe/mL (BSA-NWs_2). BSA-NWs-DOX: 9.3 μg Fe/mL and 0.25 μM of DOX (BSA-NWs-DOX_1), 28 μg Fe/mL and 0.73 μM of DOX (BSA-NWs-DOX_2). Free DOX: 0.5 μM (DOX_1) and 1 μM (DOX_2). (*p < 0.05, **p < 0.01, and ***p < 0.001).

Finally, the ability of BSA-NWs-DOX and APTES-NWs-DOX to induce cancer cell death by combining the selective drug release with the mechanical disturbance upon the application of a low frequency AMF (1 mT, 10 Hz for 10 min) was examined on MDA-MB-231 cells by Alamar Blue assay (Fig. 7).

The concentrations of NW formulations stock solutions were verified by iron ICP-MS to calculate the amount of NWs as well as the concentrations of DOX. A first important observation from Fig. 7 is that applying the AMF at the mentioned conditions to the cells without NWs did not affect the cell viability. Similarly, the cytotoxic effect produced by the free drug was not affected by the AMF at the two DOX concentrations tested (0.5 and 1 μM). Fe NWs coated with APTES or BSA did not reduce the cell viability. On the other hand, a significant cytotoxic effect emerged when the AMF was applied to cells that were incubated with NWs. A decrease of ~14% and 23% in cell viability was observed for APTES-NWs_1 (8.7 μg of Fe/mL) and APTES-NWs_2 (26 μg of Fe/mL). A higher cytotoxic effect, showing a decrease of ~21% and 28% in cell viability, was generated by BSA-NWs_1 (9.3 μg of Fe/mL) and BSA-NWs_2 (28 μg of Fe/mL) for similar amounts of Fe. These results confirm the high degree of biocompatibility of Fe NWs formulations and the possibility to kill cancer cells by magneto-mechanical stimulation at the field parameters tested. This mechanical effect is generated by the contribution of both internalized and membrane embedded NWs. It can be also suggested that BSA is a more efficient coating agent with respect to killing of cancer cells by this method.

The frequency is associated with the AMF’s amplitude and determines how fast or slow the value of the amplitude changes over time. At the frequency applied in this study (10 Hz), the change of the amplitude’s value (1 mT) is slow enough to allow the oscillatory movement of the NW. In principle, the specific loss power, which describes the power achievable per gram of magnetic material (W/g), is an increasing function of frequency and field amplitude, and represents the amount of heat that can be produced by a specific material. It is well known that the dependence of the specific loss power on frequency is linear for ferromagnetic particles^27^. For producing heating efficiency values of the orders of 10–100 W g^−1^, necessary for thermoablation, a field amplitude of ~10 kA m^−1^ and a frequency of about 100 kHz are required^26^,^45^. Moreover, as previously stated, the magneto-mechanical induction of cancer cell death with magnetic NWs has been reported with field parameters similar to those applied in this study (0.5 mT, 1 Hz and 1 kHz, 10 min) and any temperature contribution was disregarded since no change was detected between the testing groups^25^.

Most probably, internalized Fe NWs continue being oxidized and degraded after a certain time. Perez *et al*. 2016 have reported that a minimal fraction (~2%) of the dose of Ni NWs were dissolved intracellularly after 72 h and suggested that intracellular dissolution is most probably due to the acidic pH of the lysosomal compartments in the cytoplasm^42^. Based on this, although intracellular dissolution in lysosomes could also be the fate of Fe NWs, no cytotoxic contribution from NW degradation is expected during the times in which our experiments were performed.

In the case of DOX-functionalized NWs, the cytotoxic effect due to the DOX release showed a decrease of approximately 32% and 54% in cell viability for APTES-NWs-DOX_1 (8.7 μg of Fe/mL, 0.44 μM DOX) and APTES-NWs-DOX_2 (26 μg of Fe/mL, 1.3 μM DOX), respectively. For the BSA-coated NWs a decrease of 31% and 58% in cell viability was determined for BSA-NWs_1-DOX (9.3 μg of Fe/mL, 0.25 μM DOX) and BSA-NWs_2 (28 μg of Fe/mL, 0.73 μM DOX), respectively. These results show the efficacy of the selective intracellular drug release and the cytotoxic effect of the DOX. Moreover, they imply that Fe NWs are highly effective as drug carriers. Although the BSA-NWs carry less amount of DOX than the APTES-NWs, the cell viability reduction is about the same. This indicates a higher efficacy of the BSA-NWs, which might be related to a more efficient internalization as found in the confocal microscopy study.

Finally, an additive effect of the cytotoxicity was observed when the AMF was applied to cells treated with both formulations of DOX-functionalized NWs. An additional decrease of ~10% in cell viability was observed for APTES-NWs-DOX_1 (8.7 μg of Fe/mL, 0.44 μM DOX) and APTES-NWs-DOX_2 (26 μg of Fe/mL, 1.3 μM DOX), and an additional decrease of ~15% and 8% in cell viability, was observed for BSA-NWs_1-DOX (9.3 μg of Fe/mL, 0.25 μM DOX) and BSA-NWs-DOX_2 (28 μg of Fe/mL, 0.73 μM DOX). These experiments were performed using similar amounts of Fe but less amounts of DOX in the case of BSA-NWs-DOX. The synergic cytotoxic effect was statistically equal for both formulations, leading to a final decrease in cell viability of ~45% and 69% for APTES-NWs-DOX, and ~48% and 73% for BSA-NWs-DOX. Taking into account that BSA-NWs-DOX had 1.8 times less amount of DOX/Fe a significant increase in the effective cytotoxicity was detected for the BSA-NWs-DOX, which we attribute to their higher stability and cellular internalization.

In conclusion, a novel method for bimodal cancer cell destruction was developed by combining the intrinsic magneto-mechanical properties of Fe NWs coupled to the chemotoxic effect performed by an anticancer drug attached to the NWs. The Fe NWs and the two different coating agents employed, APTES and BSA, demonstrated to be highly biocompatible. The coatings also proved to be efficient for the further pH sensitive functionalization of NWs with a chemotherapeutic agent. The NWs were readily internalized and turned out to be very efficient carriers for drug delivery. The BSA-NWs formulations displayed a higher internalization degree and a broader distribution within the cells in addition to bunch in smaller and less compact clusters, making it a more efficient candidate for the induction of cell death. Furthermore, the functionalized NWs generated a large cytotoxic effect in MDA-MB-231 cells. The combination of the chemotoxic and magneto-mechanical treatment modes was found to have synergistic effects, making the proposed method an attractive option for new cancer therapies.

Future therapeutic applications would require increasing the specificity of our nanosystem. Therefore, the addition of targeting molecules such as peptides or antibodies during the functionalization step is needed for selectively directing the NWs towards target cells in further *in vivo* studies. Within this scope, using similar functionalization strategies we have shown efficient antibody targeting of nanoparticles *in vitro*^51^ and the efficacy of a thiol derivative of Nucant pseudopeptide that acts as both targeting and anticancer agent *in vitro* and *in vivo*^45^,^48^. These strategies are compatible with our system and offer great potential for future studies. Besides, due to their magnetic properties, such remotely controllable drug carriers could have advantages over other methods such as being directed to the target tissue by the application of a magnetic field thus increasing the selectivity of the system.

Compared to hyperthermia-based methods, the magneto-mechanical treatment mode requires only low field strengths and low frequencies. This is not only relevant from a safety point of view, but it also reduces the power consumption by several orders of magnitude, making it very efficient in terms of costs and technical implementation.

## Methods

The main aspects of the methods are mentioned in this section. For detailed explanations of some of the procedures see the Supplementary Information.

### Materials

APTES, BSA and DOX were purchased from Sigma-Aldrich. Polyethylene glycol-Thiol (Mw = 2000 Dalton, denoted as PEG-SH) was from Creative PEGWorks (USA). Double distilled water was used in all experiments.

### Alternating magnetic field generator

The *in vitro* alternating magnetic field (AMF) generator employed in this study is a home-made air-cooled ferrite core with a C shape and a gap of 16 mm, coiled with Litz wires. The AMF generator is part of an LCR resonant circuit allowing to independently adjusting the frequency and intensity. The core gap allows placing NUNC(TM) 4 well dishes (internal well diameter is 10 mm) for applying AMF into single well. The AMF direction is perpendicular to the wells, and its field intensity gradient is about 10% from the center of the well to the external border. Before the *in vitro* studies, we checked that the AMF generator does not heat up the cell media under the conditions employed in this study. The applied AMF was 1 mT and 10 Hz.

### Cell culture

MDA-MB-231 cell line was purchased from American Type Culture Collections (Manassas, VA, USA). MDA-MB-231 cell line was grown as monolayer in Dulbecco’s Modified Eagle’s Medium (DMEM) supplemented with 10% fetal bovine serum (FBS), 2 mM L-glutamine, 0.25 *μ*g/mL fungizone and 100 units/mL of penicillin and 100 μg/mL of streptomycin. All reagents were purchased from GIBCO. Cell lines were maintained in an incubator at 37 °C in a humidified atmosphere of 95% air and 5% CO_2_.

### Drug derivative synthesis

A DOX derivative [(5-Maleimidovaleroyl) hydrazone of Doxorubicin] was synthesized as previously described^52^, using as precursor 5-aminovaleric acid instead of 6-aminocaproic acid, to functionalize the coated NWs with DOX. ^1^H NMR (400 MHz, MeOD, δ): 7.92 (bd, lH), 7.81 (t, lH), 7.55 (d, lH), 6.57 (m, 2H), 5.51 (m, lH), 5.07 (m, lH), 4.54 (d, lH), 4.25 (m, lH), 4.06 (s, 3H), 3.61–2.7 (m, 5H), 2.55–2.26 (m, 4H), 2.20–1.90 (m, 3H), 1.62–1.25 (m, 10 H); HRMS (ESI) *m*/*z*: [M + H]^+^ calculated for C_36_H_41_N_4_O_13_, 737.2664; found, 737.2638.

### Iron nanowires synthesis

Fe NWs were fabricated by chemical electrodeposition of an iron solution into nanoporus alumina membranes. The NWs’ length of 7 μm (6.4+/−1.3 μm, SEM images) was controlled by the deposition time (1.5 h). Thereafter, the template containing the NWs was dissolved with 1 mL of 1 M NaOH in an Eppendorf tube for 20 min and the alumina membrane was removed. The NaOH solution was changed every hour for 4 times with the help of a magnetic rack (DynaMag™−2; Life Technologies, Carlsbad, CA, USA). Finally, the NWs were collected with the magnetic rack and rinsed thoroughly with ethanol for 10 to 15 times with 10-second sonication steps in between and leaving the released NWs suspended in 1 mL of absolute ethanol^40^.

### Iron nanowires characterization

The morphology, length and diameter of the NWs were investigated by scanning electron microscopy (SEM) and transmission electron microscopy (TEM) (SEM: Quanta 3D; FEI Company, Hillsboro, OR, USA; and TEM: TecnaiBioTWIN; FEI Company). Energy-dispersive X-Ray spectroscopy (EDX) was used for determining the chemical composition of NWs (scanning TEM (STEM) Tecnai BioTWIN; FEI Company). Images were analyzed using ImageJ software as previously described^25^. For TEM imaging, fresh samples were prepared in which NWs were released from the alumina template and rinsed several times with ethanol with sonication periods of 10 seconds between the washing steps. For SEM imaging sonication steps were skipped to prevent fragmentation of the NWs. Likewise, the NWs were quantified by first quantifying the amount of pores in determined area from SEM images of the alumina template and then extrapolated to the total deposition area. The amount of nanowires was obtained by taking the assumption that every pore contains a NW as previously performed^25^. The deposition area was determined using ImageJ software from where the number of NWs was obtained for further Fe mass calculation.

### Coating of iron nanowires

Fe NWs were coated with APTES and BSA through the formation of covalent bonds between the coating agent and the iron oxide (Fe_2_O_3_) interphase that surrounds the NWs formed after releasing the NWs from the alumina. The morphology of BSA and APTES coated NWs was analyzed by TEM (TecnaiBioTWIN; FEI Company).

NWs were also coated with thiol-polyethylene glycol (PEG-SH) (50 μmol PEG/g Fe). In this case, the NWs had to be first coated with APTES and further activated with 2-Iminothiolane (2-IT) and aldrithiol as previously described^53^, to finally react with thiol-PEG (2 kD).

### Functionalization of iron nanowires

For the functionalization of the coated NWs, the free primary amino groups present on APTES and BSA were first activated by the addition of 2-Iminothiolane (2-IT) for further attachment of the DOX derivative as previously explained^53^. The covalently immobilized DOXO was quantified for both, BSA and APTES formulations.

### Drug release from functionalized iron nanowires

The release of DOX from the functionalized NWs is based on the pH sensibility of the drug derivative linker. The amount of DOX released was quantified by measuring the fluorescence of free DOX released in solution (λexc = 495 nm, λem = 520–750 nm) at regular time intervals after passing 0.5 mg of functionalized NWs from PBS solution of pH 7.4 to acetate buffer solution of pH = 5.0 at 37 °C. The values were compared to the ones from a reference sample of functionalized NWs suspended in PBS solution of pH 7.4. The same process was done for APTES and BSA coated NWs.

### Functionalized iron nanowires internalization and drug release monitoring by confocal reflection microscopy

Internalization of the different Fe NWs formulations and release of DOX inside of the breast cancer cells were assessed using confocal reflection microscopy with a confocal laser scanning microscope (TCS Leica SP5) using a confocal reflection mode. Cells were treated with the NWs formulations (28 μg Fe/mL and 0.73 μM of DOX for BSA-NWs-DOX, 26 μg Fe/mL and 1.3 μM of DOX for APTES-NWs-DOX). Intracellular focal plane independent images as well as intracellular z-stacks were taken from samples incubated 24 and 72 h with NWs formulations (Supplementary Info). Z-stacks images were projected by using ImageJ software.

### Iron nanowires quantification

In order to quantify the amount of NWs that interact (get internalized or attached to the membrane) with the incubated cells, inductively coupled plasma mass spectrometry (ICP-MS) for Fe quantification was done. The quantification of Fe NWs in MDA-MB-231 cells was performed by ICP-MS, comparing different coating agents (Supplementary Info). Furthermore, the presence and distribution of Fe NWs in MDA-MB-231 cells were assessed by a Prussian blue staining assay, comparing the different coating agents (Supplementary Info).

### *In vitro* cell viability assay in the presence of functionalized iron NWs with/without a low frequency AMF

To assess cell death, cells were cultured on a 4-well plate at a density of 2.5 × 10^4^ cells per well in 500 μl of DMEM containing 10% FBS. After 24 h, the growth medium was removed and the cells were incubated for 24 h at 37 °C in the presence of different concentrations of free DOX (0.5 and 1 μM), APTES-NWs (8.7 and 26 μg Fe/mL), APTES-NWs-DOX (8.7 μg Fe/mL, 0.44 μM of DOX and 26 μg Fe/mL 1.3 μM of DOX), BSA-NWs (9.3 and 28 μg Fe/mL) and BSA-NWs-DOX (9.3 μg Fe/mL, 0.25 μM of DOX and 28 μg Fe/mL, 0.73 μM of DOX). The NWs’ concentration tested in this experiment was found by ICP-MS from the stocks. As controls, non-treated cells and empty wells were used. After incubation, cells were washed three times with PBS and then maintained in 0.5 mL of DMEM containing 10% FBS at 37 °C and 5% CO_2_ incubator. Then, after 24 h, the magnetic field was applied for 10 minutes maintaining a temperature of 37 °C. A homemade equipment described before was used for the generation of an electromagnetic field with a strength (amplitude) of 1 mT and a frequency (υ) of 10 Hz. After 72 h of post-incubation, the medium was replaced with of DMEM containing 10% FBS and 10% of Resazurin dye (1 mg/ml PBS). Cells were maintained at 37 °C and 5% CO_2_ incubator for 6 h and then, a Synergy H4 microplate reader was used to determine the amount of Resazurin by measuring the absorbance of the reaction mixture (excitation 540 nm, emission 590 nm). 600 μl of 10% of Resazurin dye was added to empty wells as a negative control. The viability of the cells was expressed as the percentage of absorption of treated cells in comparison with control cells (without NWs). All experiments were carried out in quadruplicates. All the data obtained were plotted and statistically analyzed using the software package GraphPad Prism version 5.0 for Windows. All samples were compared using a one-way ANOVA and Bonferroni post-hoc test ( ^*^P < 0.05, ^**^P < 0.01, and ^***^P < 0.001). Only significant differences among the samples are indicated in the charts.

## Additional Information

**How to cite this article**: Martínez-Banderas, A. I. *et al*. Functionalized magnetic nanowires for chemical and magneto-mechanical induction of cancer cell death. *Sci. Rep*. **6**, 35786; doi: 10.1038/srep35786 (2016).

## Supplementary Material

Supplementary Information

Supplementary Movie S1

Supplementary Movie S2
