# Supplementary material for: Functionalized magnetic nanowires for chemical and magneto-mechanical induction of cancer cell death
Source: Sci Rep. 2016 Oct 24;6:35786. doi: 10.1038/srep35786 (PMC5075884; doi:10.1038/srep35786)
Supplement: Supplementary Information [file srep35786-s1.pdf]

Supplementary Information

Functionalized magnetic nanowires for chemical and magneto-mechanical induction of cancer cell death

Aldo Isaac Martínez-Banderas[1,+], Antonio Aires[2,3,+], Francisco J. Teran[2], Jose Efrain Perez[1], Jael F. Cadenas[4], Nouf Alsharif[1], Timothy Ravasi[1], Aitziber L. Cortajarena[2,3,4,]*, Jürgen Kosel[5,]*

[1]Division of Biological and Environmental Sciences and Engineering, King Abdullah University of Science and Technology. Thuwal Jeddah, 23955-6900, Saudi Arabia.

[2]IMDEA Nanociencia and Nanobiotechnology Unit associated to Centro Nacional de Biotecnología (CNB-CSIC), Campus Universitario de Cantoblanco, Madrid, 28049, Spain.

[3]CIC BiomaGUNE, Parque Tecnológico de San Sebastián, Paseo Miramón 182, Donostia-San Sebastián 20009, Spain.

[4]Ikerbasque, Basque Foundation for Science, Mª Díaz de Haro 3, E-48013 Bilbao, Spain

[5]Division of Computer, Electrical and Mathematical Sciences and Engineering, King Abdullah University of Science and Technology. Thuwal Jeddah, 23955-6900, Saudi Arabia.

[*]Corresponding Authors. E-mail: jurgen.kosel@kaust.edu.sa and aitziber.lopezcortajarena@imdea.org, alcortajarena@cicbiomagune.es

[+] These authors contributed equally to this work.

Methods

Iron nanowires synthesis

A 99.99 % pure aluminum substrate (Goodfellow, London, UK) was cleaned with acetone, isopropanol and deionized water followed by an electropolishing process for evening its surface. A two-step anodization of the polished aluminum was carried out with 0.3 M oxalic acid at 4°C applying a voltage of 40 V to a sealed cell containing the Al film applying constant stirring, which resulted in the growth of a porous anodic alumina template with hexagonally highly ordained nanopores with diameters from 30 to 40 nm. The first anodization process lasted for 24 h creating set of random pores whereas that the second one had a duration of 4 h, yielding pores with parallel orientation. Between the anodization processes, the cell was washed with deionized water and then filled with an aqueous solution of 0.4 M H_3PO_4 and 0.2 M CrO_3 to remove the alumina layer at a temperature of 30 ºC and constant agitation. Dendrites were synthesized in order to connect the bottom of the pores with the Al for the electrodeposition of a Fe solution, allowing the flow of current. Fe NWs were grown into the alumina template by pulsed electrodeposition with current pulses limited to 60 mA employing a solution composed of 0.5 M $FeSO_4(7H_2O)$, 0.5 M Na_2SO_4, 0.4 M HBO_3 and 0.1g/100 mL of ascorbic acid.

Coating of iron nanowires

Fe NWs were coated with APTES and BSA through the formation of covalent bonds between the coating agent and the iron oxide (Fe_2O_3) interphase that surrounds the NWs.

a) APTES coating of iron nanowires

Fe NWs were coated with (3-aminopropyl)triethoxysilane (APTES) by adding 0.0946 g of APTES per each 100-300 mg of Fe to the NWs suspended in ethanol with a final volume of 5 mL in a falcon tube and sonicating for 1 h. Since basic catalysis is required for the reaction, 200

μL of milliQ water and 10 μL of NaOH 1 M were added, followed by a second 1 h sonication process. Finally, the NWs were washed 5 times with 1 mL absolute ethanol and stored at room conditions in an Eppendorf tube. For further functionalization steps, APTES coated NWs were washed 3 times with phosphate buffer (PB), 10 mM and pH 7.4, in sterile conditions.

b) BSA coating of iron nanowires

For bovine serum albumin (BSA) coating, NWs were suspended in PB, 10 mM and pH 7.4, to a final volume of 2.5 mL in a glass vial and added with 0.8 mg of BSA/mg of Fe, followed by 1.5 h of sonication. The BSA coated NWs (BSA-NWs) were washed 3 times with PB and stored at 4°C in sterile conditions. The amount of albumin attached to the NWs was determined from Bradford assay of the supernatant containing BSA that remained in solution after the coating process.

The morphology of BSA and APTES coated NWs was analyzed by transmission electron microscopy (TEM) (TEM: TecnaiBioTWIN; FEI Company). For electron imaging, fresh samples were prepared in which NWs were released from the alumina template and rinsed several times with ethanol with sonication periods of 10 seconds in between the washes followed by the respective coating methods for each sample.

Functionalization of iron nanowires

a) Activation of coated nanowires

For the functionalization of the coated NWs, the free primary amino groups present on APTES and BSA were activated by the addition of 2-Iminothiolane (2-IT) at a concentration of 500 μmol/g of Fe that adds thiol moieties (sulfhydryl groups) for further attachment of the drug, which was previously modified for the addition of a sulfhydryl group. The activation of the coated NWs was evaluated by quantifying the content of the sulfhydryl groups via the addition

of aldrithiol, a molecule composed of two pyridines joined by a disulfide bond. The free sulfhydryl of the activated NWs reacts with the disulfide bound of the aldrithiol, releasing a 2-pyridinethione that was quantified (λmax = 343 nm, $\varepsilon_{343\ nm}$ = 8080 L mol^{-1} cm^{-1}) with a UV/Vis and fluorescence spectrophotometer Synergy H4 microplate reader (BioTek) using 96-well plates as previously described.[47] The activation process was done in sterile conditions.

b) Drug attachment to activated BSA and APTES-nanowires

Activated BSA and APTES coated NWs were suspended in 1 mL of phosphate buffered saline (PBS) of pH 7.4. DOX derivative 1 mM in DMF was added to reach 80 μmol of DOX/g Fe. The sample was incubated overnight at 37 °C in slow oscillation to maintain the NWs in suspension. Functionalized NWs were retained with a magnetic rack and washed 3 times with PBS solution. From the functionalization supernatant, the covalently immobilized DOX onto thiolated NWs was indirectly determined by quantification of free DOX in solution (λmax = 495 nm) by UV/Vis spectrophotometry and comparing the result with the result obtained from a solution of free DOX at the same concentration. All functionalization processes were carried out under sterile conditions and the DOX solution used was filtered through a 0.22-μm strainer.

Functionalized iron nanowires internalization and drug release monitoring by confocal reflection microscopy

Two 4-well plates were added with cover slips and seeded with 25000 cells/well and incubated for 24 h to reach confluence in the mentioned conditions. Cells were treated with the NWs formulations and incubated for 24 h and 72 h at 37 ºC, 5% CO_2. Cells were washed twice with PBS and fixed with 500 µL/well of the solution containing 4% of paraformaldehyde and 0.5% of triton 100x for 5 minutes at room conditions followed by a second incubation period of 10-15 minutes with a solution containing only 4% paraformaldehyde removing first the previous

solution. Thereafter, the fixing media was removed and the cells incubated with 500 µL of DAPI 300 nM for 5 minutes covering the plates from light. Finally, cells were washed twice with PBS solution, dried at room conditions and put on microscope slides with Fluoroshield™ for observation with a TCS Leica SP5 confocal laser scanning microscope (CLSM) using a confocal reflection mode.

Iron nanowires quantification

a) Inductively coupled plasma mass spectrometry

40000 cells/well were seeded and incubated for 24 h to reach confluence in the mentioned conditions. The cells were treated with NWs formulations (26 µg Fe/mL, 1.3 µM of DOX for APTES-NWs-DOX and 28 µg Fe/mL, 0.73 µM of DOX for BSA-NWs-DOX and incubated for 24 h at 37 ºC, 5% CO_2. The cells were washed twice with PBS and incubated for 10-15 min with 500 µL of Trypsin at 37 ºC. Unattached cells of each treated group were collected in Eppendorf tubes and counted by bright field microscopy in a Neubauer chamber. The Eppendorf tubes were centrifuged at 10000 rpm for 20 min and the supernatant discarded carefully. 300 µL of 37% HCl was added to the cell pellet and the resultant suspension was sonicated for 30 minutes at 40 ºC. Finally, 2700 mL of bi-distilled water was added. The Fe concentration was determined by measuring the sample using an ICP-MS, NexION 300XX (Perkin Elmer) (n = 4). The Fe concentration in stocks solutions of Fe NWs formulations was also quantified.

b) Prussian blue staining assay

40000 cells/well were seeded in cover slips put in a 24 well and incubated for 24 h at 37 ºC, 5% of CO_2 to reach confluence. The incubated cells were treated separately with non-coated Fe NWs, APTES-NWs, APTES-NWs-PEG and BSA-NWs. Before the treatment, NWs were washed 3 times with PBS and suspended in fresh medium in sterile conditions. Each one of these

samples of Fe NWs was added with an amount of 0.01 mg Fe/well in duplicates and incubated for 24 h more at 37 ºC and 5% CO_2. After this second incubation period, the cells were washed with PBS three times, fixed with paraformaldehyde and 1 mL of 4% potassium ferrocyanide ($Fe(CN)_6^{4-}$) was added together with 1 mL of 4% HCl for the acid catalysis (all PanreacQuímica) and incubated for 30 min at room conditions. Thereafter, each well was washed 3 times with milliQ water and counter stained with Neutral Red 0.5% (2-5 min). Finally the cover slips were washed 3 times with milliQ water, taken out from the 24 wells plate and dried at room conditions for 1 h. Once dried, the cover slips containing the cells were put over microscope slides adding depex mounting media (Sigma-Aldrich) for further observation in bright field microscopy.

Supplementary Figures

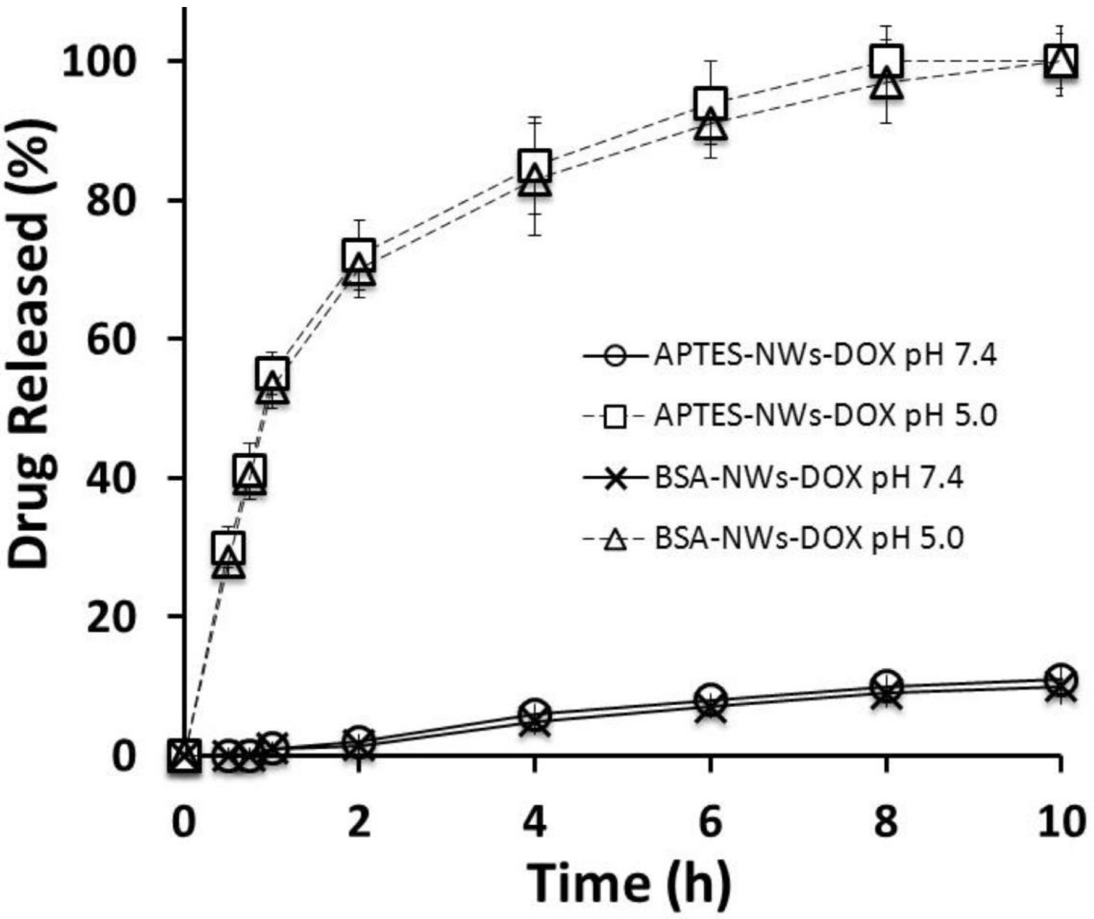

Figure S1. Release kinetics of DOX from APTES-NWs-DOX and BSA-NWs-DOX. With pH 7.4, black triangles, empty circles and solid line; pH 5.0, empty triangles, black circles and dashed line.

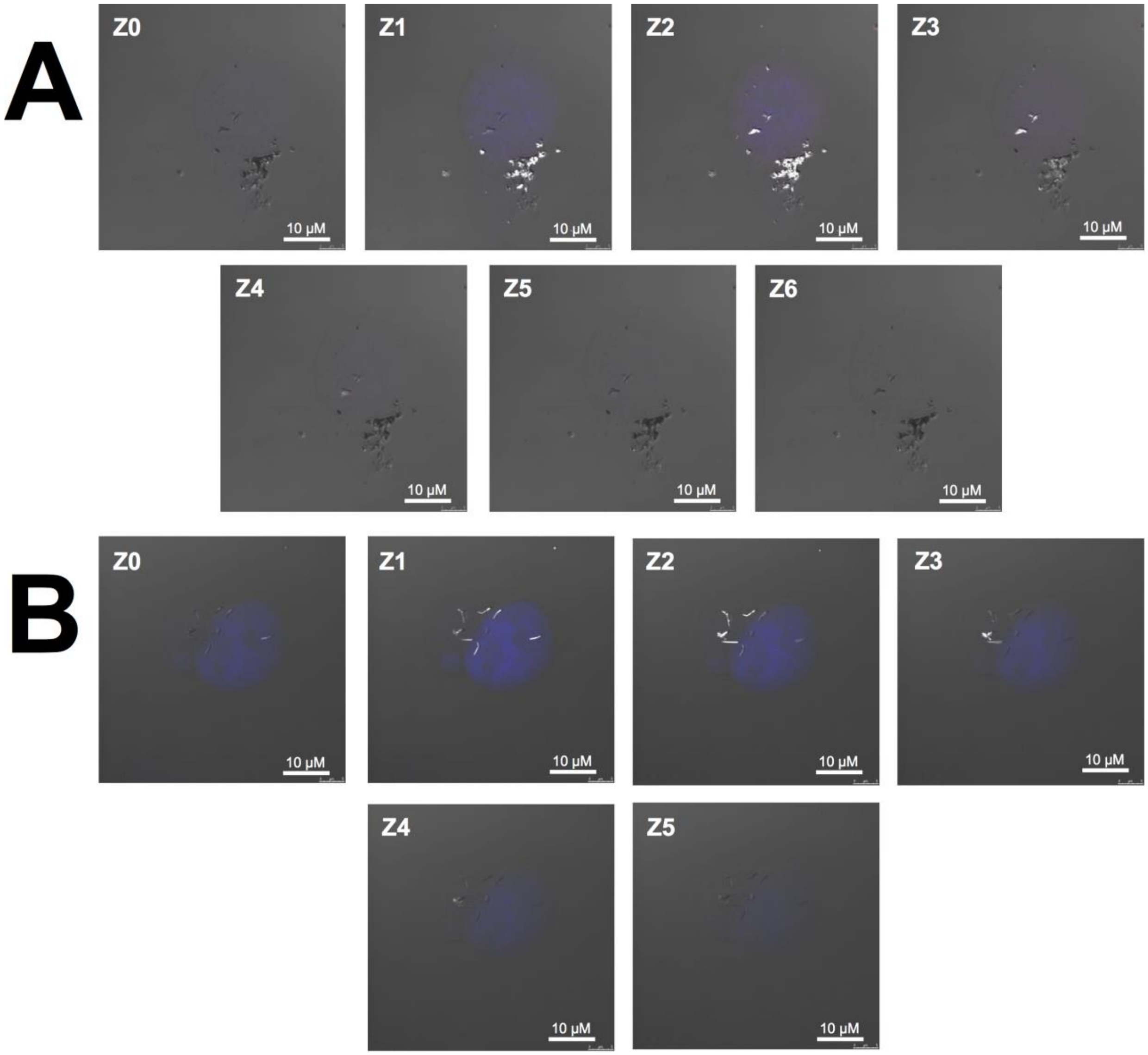

Figure S2. Z-stack images of a MDA-MB-231 cell treated with **A**. APTES-NWs-DOX and **B**. BSA-NWs-DOX after 24 h of incubation. Scale bar is 10 µm.

Supplementary Videos

Video S1. Z-stack projection of a MDA-MB-231 cell treated with APTES-NWs-DOX after 24 h of incubation.

Video S2. Z-stack projection of a MDA-MB-231 cell treated with BSA-NWs-DOX after 24 h of incubation.
